# Heat shock factor 1 is a potent therapeutic target for enhancing the efficacy of treatments for multiple myeloma with adverse prognosis

**DOI:** 10.1186/s13045-015-0135-3

**Published:** 2015-04-23

**Authors:** Sophie Bustany, Julie Cahu, Géraldine Descamps, Catherine Pellat-Deceunynck, Brigitte Sola

**Affiliations:** Normandie Univ, UNICAEN, EA4652, Caen, France; CRCNA, INSERM U892, CNRS UMR6299, Université de Nantes, Nantes, France

**Keywords:** Myeloma, Heat shock proteins, HSP inhibitor, Heat shock factor 1, Combined therapy, Lenalidomide, Dexamethasone, Bortezomib, Combination index, Apoptosis

## Abstract

Deregulated expression of heat shock proteins (HSPs) encoding genes is frequent in multiple myeloma. HSPs, which are molecular chaperones involved in protein homeostasis pathways, have emerged recently as promising therapeutic targets. Using human myeloma cell lines and primary myeloma cells belonging to various molecular groups, we tested the efficacy of HSP90, HSP70, and heat shock factor 1 (HSF1) inhibitors alone or associated with current antimyeloma drugs. We report here that KNK-437 (an inhibitor of HSF1) and bortezomib have additive effects on apoptosis induction in cells belonging to groups with bad prognosis.

**Electronic supplementary material:**

The online version of this article (doi:10.1186/s13045-015-0135-3) contains supplementary material, which is available to authorized users.

## Findings

Deregulated expression of heat shock proteins (HSPs) and heat shock transcription factor 1 (HSF1) plays a major role in the pathogenesis of multiple myeloma (MM) [1,2]. In turn, several HSP/HSF1 inhibitors are currently undergoing preclinical and/or clinical investigations [3,4].

**Figure 1:**
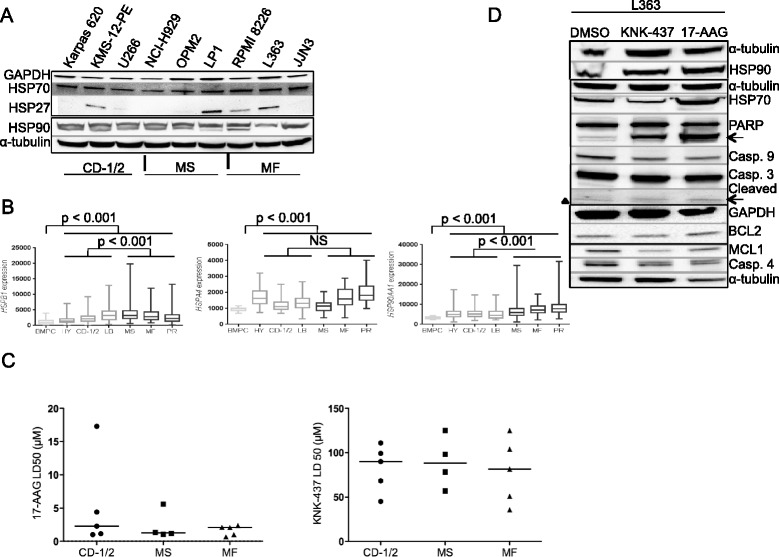
HSP90 and HSF1 inhibitors are potent antimyeloma drugs. **(A)** HSP expression was determined in a panel of HMCLs belonging to CD-1/2, MS, and MF groups ([5] and Additional file 2) by Western blotting. Blots were incubated with the following Abs: anti-HSP27, −HSP70, and -HSP90 from Santa Cruz Biotech.; anti-GAPDH from Life Technologies; and α-tubulin from Dako. Abs anti-GAPDH and -α-tubulin served for gel loading control. **(B)** Affymetrix gene expression profiles of purified myeloma cells (Additional file 1). *HSP90AA1*, *HSPB1*, and *HSPA4* gene expressions (in Affymetrix signal units) are indicated for each patient in the different molecular groups according to [6]: HY, CD-1/2, LB corresponding to standard risk in light gray, MS, or MF, and PR corresponding to high risk in dark gray. The expression of those genes was also analyzed in normal bone marrow plasma cells (BMPC). *p* < 0.001 and NS (not significant) with Student’s *t* test. **(C)** The cell lines used in this assay are described in Additional file 1. Cells (0.5 × 10^6^/ml) were seeded for 48 h in 96-well plates and treated with serial dilutions of 17-AAG (20 to 0.3 μM) or KNK-347 (200 to 3.1 μM). Cell death was then assessed using flow cytometry with the combined analysis of APO2.7 (Beckman Coulter) staining according to the manufacturer’s recommendation and the altered cellular morphology characteristics of apoptosis (lower FSC-H and higher SSC-H). Flow cytometry analysis was performed on a FACSCalibur using the CellQuest software (BD Biosciences). The LD50 was defined as the concentration that killed 50% of cells (mean of 3 experiments). **(D)** L363 cells were treated for 24 h with 100 μM KNK-437 or 5 μM 17-AAG. Western blots were obtained as before. Ab anti-MCL1 was obtained from Santa Cruz Biotech. and anti-BCL2 from Dako (Glostrup, Denmark). The cleaved forms of PARP and procaspase 3 are arrowed. ▲ marked a non-specific band.

We used human myeloma cell lines (HMCLs) belonging to several molecular groups [5,6] to analyze HSP expression (Figure 1A). HSP90 and its co-chaperone HSP70 were constitutively expressed in all HMCLs. HSP27 expression was more heterogeneous. Using the Little Rock public database [6], we investigated whether the expression of *HSPB1*, *HSPA4*, and *HSP90AA1* genes varied according to the MM molecular classification. Compared to normal bone marrow plasma cells, *HSP* genes were constantly overexpressed (Figure 1B). *HSPB1* and *HSP90AA1* expressions were higher in the groups with bad prognosis, PR/MS/MF, and *HSPA4* expression in the HY/MF/PR groups. The material and methods used in the study are detailed in Additional file 1.

We studied the sensitivity of HMCLs towards 17-AAG that targets HSP90 or KNK-437 (an inhibitor of HSF1 and, in turn, of both HSP70 and HSP27). HMCLs were constantly sensitive to both inhibitors although heterogeneously responding (Figure 1C, Additional files 2 and 3). This suggests that inhibiting HSPs might potentiate drug treatments for MM patients.

HSPs contribute to MM survival by impairing the mitochondria- and endoplasmic reticulum (ER)-mediated apoptotic pathways [7,8]. In L363 cells (MF group), HSP70 expression decreased following KNK-437 treatment while increased after 17-AAG (Figure 1D). As confirmed by the activation of procaspases 9 and 3 and the cleavage of PARP, a mitochondrial-mediated apoptosis was triggered. The expression of anti-apoptotic BCL2 and MCL1 proteins decreased after KNK-437 treatment. Last, both inhibitors induced a decrease of the procaspase 4, thus favoring an ER stress.

**Figure 2:**
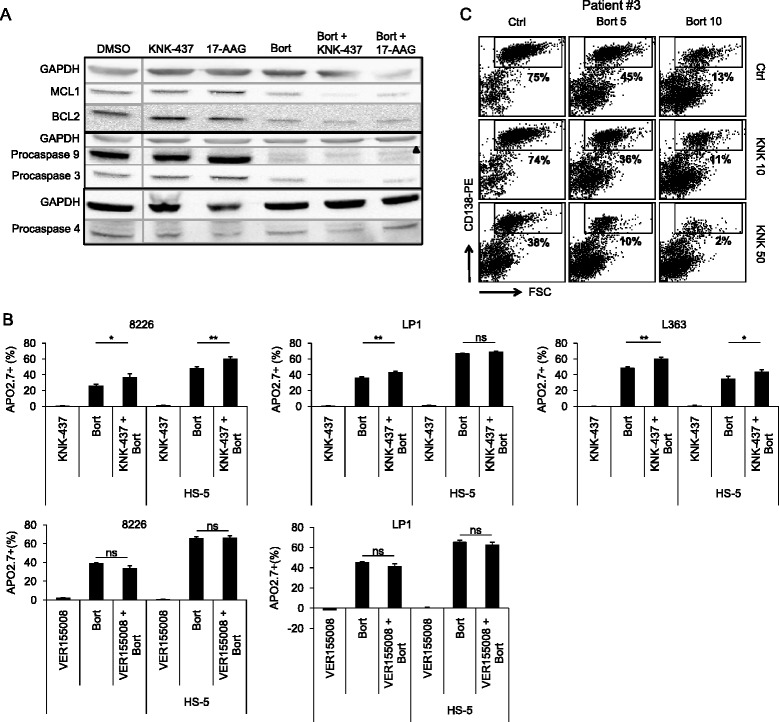
Inhibitors of HSP90 and HSF1 co-operate differently with antimyeloma drugs. **(A)** LP1 MM cells were treated with 10 μM KNK-437 or 100 nM 17-AAG or/and 10 nM bortezomib. Whole cell extracts were analyzed as before by Western blots with the indicated Abs. Anti-GAPDH Ab controlled gel loading. ▲ marked an unspecific band. **(B)** L363, LP1, and 8,226 cells were cultured on HS-5 cells 24 h before being treated as previously, stained with anti-APO2.7-PE recognizing specifically apoptotic cells followed by flow cytometry analysis (Gallios, Beckman Coulter). Means and SD of three independent experiments are presented in histograms. *p < 0.05, **p < 0.01, ns, not significant with Student’s *t* test. **(C)** Primary cells from patient #3 were treated with vehicle or bortezomib (5 or 10 nM) or KNK-437 (10 or 50 μM) for 24 h and then analyzed for CD138 labeling (FL2) as described [10]. Cell death was determined by the percentage of CD138+ cells that have lost CD138 expression. The percentage of living cells (CD138+) for each culture condition is indicated on the graph. At least 2 × 10^4^ events were gated for each culture condition with the FACsCalibur cytometer; data were analyzed with the CellQuest software.

We investigated the capacity of HSP90/HSF1 inhibitors to co-operate with common antimyeloma drugs (bortezomib, dexamethasone, or lenalidomide). We calculated the combination index using the method of Chou [9]. Both inhibitors antagonized lenalidomide effects, suggesting that those associations could be harmful (Additional file 4). The combination of KNK-437 with bortezomib or dexamethasone was highly potent in all cell lines tested but not the association 17-AAG/dexamethasone. The activation of procaspases 9/3 and the decrease of MCL1 and BCL2 levels were enhanced by the association KNK-437/bortezomib but not the association 17-AAG/bortezomib (Figure 2A). VER-155008, a strict HSP70 inhibitor, combined with bortezomib was no more potent for inducing apoptosis (Figure 2B).

We tested the response of HMCLs co-cultured with human bone marrow stromal cells (HS-5 cells). The percentage of apoptotic cells was enhanced by the co-treatment KNK-437/bortezomib (Figure 2B). This indicates that KNK-437/bortezomib combined therapy could overcome cell adhesion-mediated drug resistance.

We finally analyzed the response of primary cells isolated from four MM or plasma cell leukemia (PCL) patients (Additional file 5) towards KNK-437 and bortezomib after CD138 staining [10]. For patient #3, the fraction of CD138+ cells decreased in the presence of both drugs, revealing an additive effect in primary cells (Figure 2C). Similar results were obtained for other MM primary samples (Additional file 6).

Our results strongly suggest that HSF1 inhibitors might be promising agents in association with bortezomib-based therapeutic protocols to treat MM patients with adverse prognosis or in relapse.

## Additional files

Additional file 1:
**Material and methods used in the study.** The file contains data on cell line cultures, treatments, and proliferation measurement; primary samples, treatments, and CD138 expression analyses; and gene expression profiling. Additional file 2:
**Response towards HSP90 and HSF1 inhibitors in a panel of MM cell lines.** Cells (0.5 × 10^6^ cells/ml) were seeded for 48 h in 96-well plates and treated with increasing concentrations of 17-AAG (0.3 to 20 μM) or KNK-347 (3.1 to 200 μM). Cell death was measured by APO2.7 staining and cytometry sorting. LD50 values were defined as the dose that killed 50% of cells. Data represent the mean and SD of three experiments. Additional file 3:
**Inhibitors of HSP90 and HSF1** **co-operate** **differently with antimyeloma drugs in various HMCLs.** Cells were treated for 24 h with HSP inhibitors and then with dexamethasone (Dex), bortezomib (Bort) or lenalidomide (Len) for additional 24 h at the concentrations indicated or with vehicle (DMSO). The absorbance (OD at 490 nm) of each clone treated with the drug is expressed relative to that of the corresponding clone treated with vehicle (ratio defined as 1 arbitrary unit, AU). For each set of culture conditions, the mean of triplicate ratios is indicated on the graph, together with the SD. Additional file 4:
**Interactions between the drugs analyzed by the combination index (CI) method.** HMCLs were treated for 24 h with HSP90 or HSF1 inhibitors and with dexamethasone, bortezomib, or lenalidomide for additional 24 h at the indicated concentrations. Cell viability was then determined by MTT assay. CIs were calculated according to Chou [9]. CI < 1 indicates synergy; CI = 1, additive effect; CI > 1, antagonism (gray boxes). Additional file 5:
**Clinical characteristics of MM/PCL patients.** Samples were obtained from patients at diagnosis (D) or relapse (R); patients had multiple myeloma (MM), primary plasma cell leukemia (pPCL), or secondary plasma cell leukemia (sPCL). Additional file 6:
**Additivity of bortezomib and** **KNK-347** **co-treatment** **on MM primary samples.** Primary cells were obtained from patients with MM or PCL. Purified CD13+ cells were cultured for 24 h and then treated with 5 nM bortezomib alone or in combination with 10 μM KNK-437. Cell death was determined as the percentage of CD138+ cells that have lost CD138. The percentage of dead cells directly measured (observed) and the percentage of dead cells calculated for an additive effect (expected) were not significantly different (*p* = 0.37, Wilcoxon matched-pairs signed-rank test). This signifies that the effect of the combination of both drugs was indeed additive.

## Abbreviations

Ab: Antibody
BCL2: B-cell lymphoma 2
BMPC: Normal bone marrow plasma cells
CI: Combination index
HMCL: Human myeloma cell line
HSF1: Heat shock transcription factor 1
HSP: Heat shock protein
MCL1: Myeloid cell leukemia 1
MM: Multiple myeloma
PARP: Poly (ADP-ribose) polymerase
VER-155008: 5′-*O*-[(4-Cyanophenyl)methyl]-8-[[(3,4-dichlorophenyl)methyl]amino]-adenosine
17-AAG: 17-Allylamino-17-demethoxy-geldanamycin
