# Supplementary material for: Heat shock factor 1 is a potent therapeutic target for enhancing the efficacy of treatments for multiple myeloma with adverse prognosis
Source: J Hematol Oncol. 2015 Apr 23;8:40. doi: 10.1186/s13045-015-0135-3 (PMC4435646; doi:10.1186/s13045-015-0135-3)
Supplement: Additional file 1: — Material and methods used in the study. The file contains data on cell line cultures, treatments, and proliferation measurement; primary samples, treatments, and CD138 expression analyses; and gene expression profiling. [file 13045_2015_135_MOESM1_ESM.docx]

**Additional File 1**

**Material and Methods**

***Cell line cultures, treatments and proliferation measurement***

The human bone marrow stromal cell line HS-5 was purchased from ATCC® (CRL-11882). RPMI 8226 (here 8226), Karpas 620, U266, NCI-H929, OPM2, NAN1, NAN3, NAN7, MDN, XG1, XG5, XG6, L363, JIM3, MM.1S, and KMS12PE MM cells have been described previously [1]. LP1 cells were a generous gift of Dr D. Bouscary (Institut Cochin, Paris, France), MM.1S cells were kindly provided by Dr S. Rosen (Lurie Hospital, Chicago, IL, USA and JIM3 by Dr I. MacLennan (University of Birgmingham, UK). At their arrival, their identity was checked by karyotyping and surface markers expression; cells were then amplified and frozen. JJN3 cell line was purchased from DSMZ (ACC-541, Braunschweig, Germany). HS-5 and human myeloma cell lines (HMCLs) were maintained in RPMI 1640 medium (Lonza, Basel, Switzerland) supplemented with 2 mM L-glutamine (Lonza), 10% fetal calf serum (PAA Laboratories, Villacoublay, France) and antibiotics (Lonza). The interleukin 6 (IL6)-dependent cells (XG1, XG5, XG6, NAN1, NAN3 and NAN7) were cultured in medium supplemented with 3 ng/ml recombinant IL6 as described [1]. Cell viability was determined by direct cell counting after trypan blue staining. Cell proliferation was quantified using CellTiter 96® AQueous One Solution (MTT [(3-(4,5)-dimethylthiazol-2-yl)-2,5-diphenyltetrazolium bromide] assay (Promega, Charbonnières, France) according to the supplier’s instructions.

***Primary samples, treatments and CD138 expression analyses***

After informed consent, blood or bone marrow samples from patients with MM or plasma cell leukemia (PCL) were collected at the Department of Hematology at the University Hospital of Nantes or at the Intergroupe Francophone du Myélome (ethical approval n° DC-2011-1399). The clinical features of MM and PCL patients are presented in Additional File 4. Plasma cells were purified using CD138 immunomagnetic beads (Stemcell Technologies, Le Plessis Robinson, France). Deletion of the short arm of chromosome 17 (del17p) and t(4;14) were assessed by FISH as described [2]. Purified CD138+ cells were cultured for 24 h in RPMI 1640 containing 5% FCS and 3 ng/ml rIL6, then treated or not with 5-10 nM bortezomib alone or in combination with 10-50 µM KNK-437. Cell death was measured *via* the loss of CD138 staining as described previously [3].

***Gene expression profiling***

Affymetrix data from a cohort of 414 untreated patients from the Arkansas Cancer Research Center were analyzed for *HSP90AA1*, *HSPA4* and *HSPB1* expression (211968_s_at, 211015_s_at and 201841_s_at probes respectively) using the Amazonia! database [4].

**References**

1. Moreaux J, Klein B, Bataille R, Descamps G, Maïga S, Hose D, Goldschmidt H, Jauch A, Rème T, Jourdan M, Amiot M, Pellat-Deceunynck C: A high-risk signature for patients with multiple myeloma established from the molecular classification of human myeloma cell lines. *Haematologica* 2011, 96:574-582.

2. Lodé L, Eveillard M, Trichet V, Soussi T, Wuillème S, Richebourg S, Magrangeas F, Ifrah N, Campion L, Traullé C, Guilhot F, Caillot D, Marit G, Mathiot C, Facon T, Attal M, Harousseau JL, Moreau P, Minvielle S, Avet-Loiseau H: Mutations in TP53 are exclusively associated with del(17p) in multiple myeloma. *Haematologica* 2010, 95:1973-1976.

3. Surget S, Chiron D, Gomez-Bougie P, Descamps G, Ménoret E, Bataille R, Moreau P, Le Gouill S, Amiot M, Pellat-Deceunynck C: Cell death via DR5, but not DR4, is regulated by p53 in myeloma cells**.** *Cancer Res* 2012, 72:4562-4573

4. Le Carrour T, Assou S, Tondeur S, Lhermitte L, Lamb N, Rème T, Pantesco V, Hamamah S, Klein B, De Vos J: Amazonia!: An online resource to Google and visualize public human whole genome expression data**.** *Open Bioinforma J* 2010, 4:5-10. (<http://amazonia.transcriptome.eu>).
