# Supplementary material for: Heat shock factor 1 is a potent therapeutic target for enhancing the efficacy of treatments for multiple myeloma with adverse prognosis
Source: J Hematol Oncol. 2015 Apr 23;8:40. doi: 10.1186/s13045-015-0135-3 (PMC4435646; doi:10.1186/s13045-015-0135-3)
Supplement: Additional file 2: — Response towards HSP90 and HSF1 inhibitors in a panel of MM cell lines. Cells (0.5 × 10^6^ cells/ml) were seeded for 48 h in 96-well plates and treated with increasing concentrations of 17-AAG (0.3 to 20 μM) or KNK-347 (3.1 to 200 μM). Cell death was measured by APO2.7 staining and cytometry sorting. LD50 values were defined as the dose that killed 50% of cells. Data represent the mean and SD of three experiments. [file 13045_2015_135_MOESM2_ESM.docx]

**Additional File 2**

**Response towards HSP90 and HSF1 inhibitors in a panel of MM cell lines**

| Cell line | Classification | 17-AAG  LD50 (μM) | KNK-437  LD50 (μM) |
| --- | --- | --- | --- |
| KMS-12-PE | CD-1/2 | 1.00 ± 0.87 | 68.3 ± 36.2 |
| MDN | CD-1/2 | 1.14 ± 0.57 | 45.1 ± 6.5 |
| XG1 | CD-1/2 | 4.40 ± 1.70 | 90.0 ± 5.0 |
| XG5 | CD-1/2 | 17.30 ± 3.90 | 11.0 ± 5.0 |
| NAN7 | CD-1/2 | 2.27 ± 1.42 | 78.3± 17.6 |
| NCI-H929 | MS | 1.33 ± 0.29 | 99.3± 21.1 |
| NAN3 | MS | 1.06 ± 0.11 | 56.9± 9.5 |
| JIM3 | MS | 5.60 ± 0.70 | 125.0± 10.0 |
| OPM2 | MS | 1.17 ± 0.29 | 98.3 ± 22.5 |
| JJN3 | MF | 1.03 ± 0.40 | 125.0 ± 35.4 |
| NAN1 | MF | 2.38 ± 0.24 | 35.8 ± 4.2 |
| MM.1S | MF | 2.10 ± 0.80 | 104.0 ± 12.0 |
| XG6 | MF | 2.13 ± 0.24 | 81.5 ± 28.3 |
| 8226 | MF | 0.70 ± 0.30 | 51.0 ± 14.0 |

Cells (0.5 x 10^6^ cells/ml) were seeded for 48 h in 96-well plates and treated with increasing concentrations of 17-AAG (0.3 to 20 μM) or KNK-347 (3.1 to 200 μM). Cell death was measured by APO2.7 staining and cytometry sorting. LD50 values were defined as the dose that killed 50% of cells. Data represent the mean and SD of three experiments.
