# Supplementary figures and images for: Heat shock factor 1 is a potent therapeutic target for enhancing the efficacy of treatments for multiple myeloma with adverse prognosis
Source: J Hematol Oncol. 2015 Apr 23;8:40. doi: 10.1186/s13045-015-0135-3 (PMC4435646; doi:10.1186/s13045-015-0135-3)

A

8226

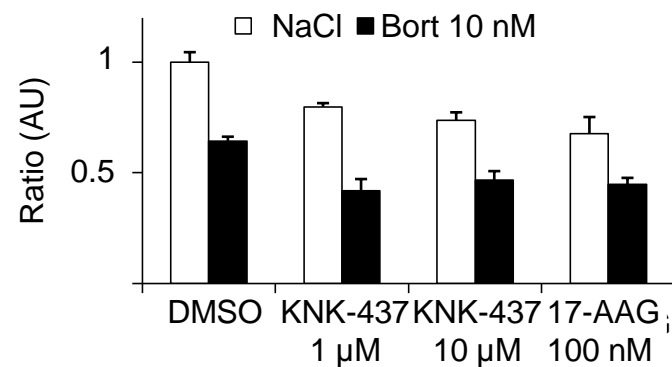

L363

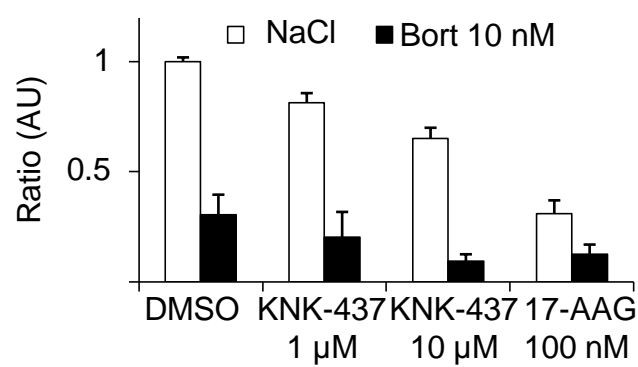

LP1

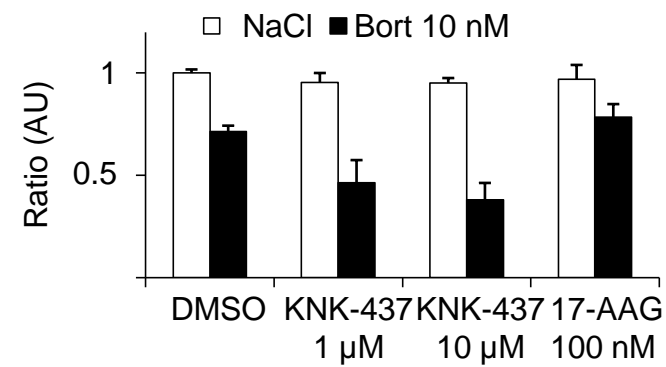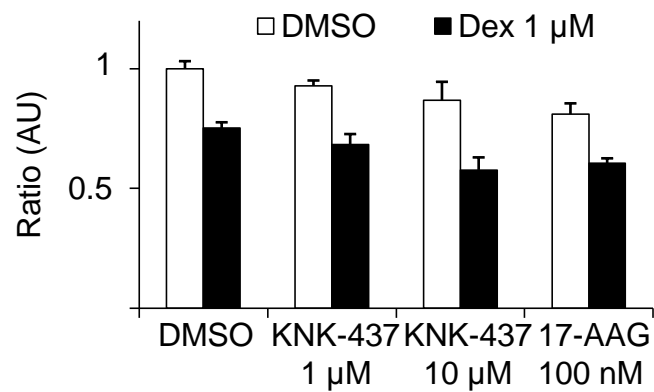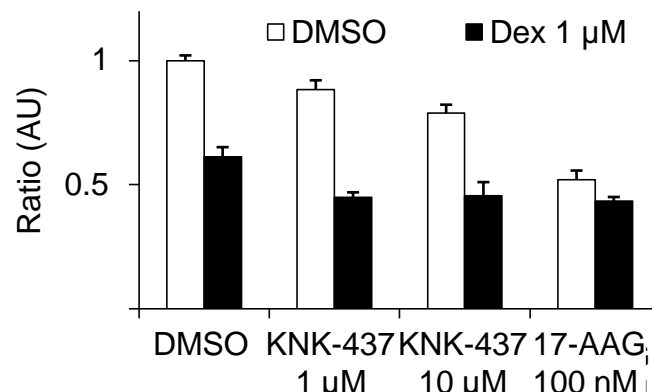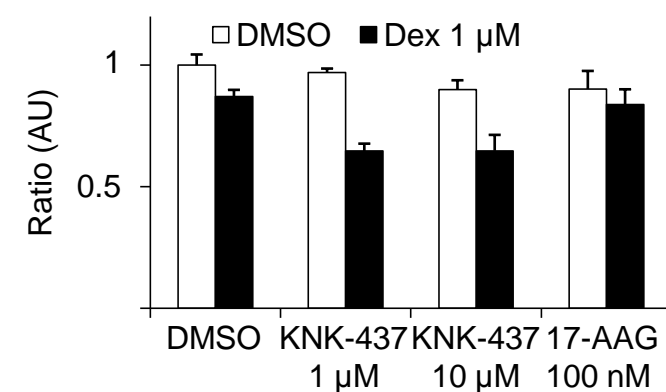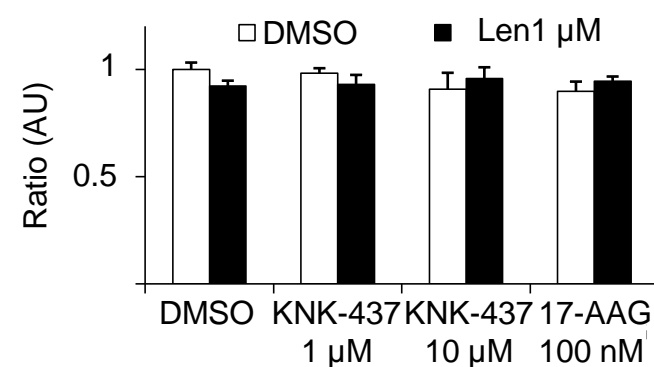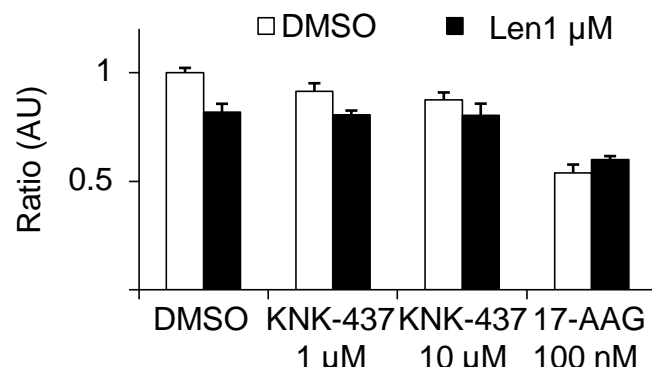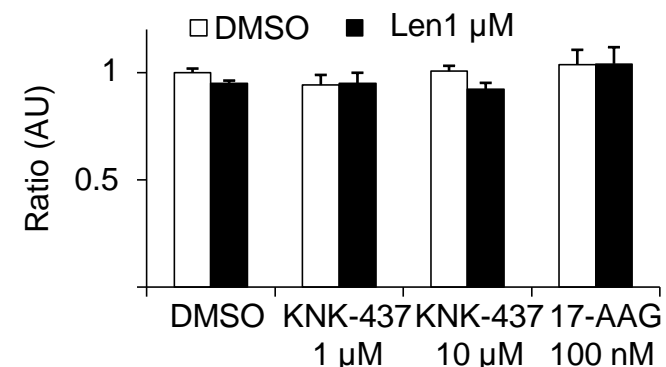

Supplementary Figure 1

Supplement: Additional file 3: — Inhibitors of HSP90 and HSF1 co-operate differently with antimyeloma drugs in various HMCLs. Cells were treated for 24 h with HSP inhibitors and then with dexamethasone (Dex), bortezomib (Bort) or lenalidomide (Len) for additional 24 h at the concentrations indicated or with vehicle (DMSO). The absorbance (OD at 490 nm) of each clone treated with the drug is expressed relative to that of the corresponding clone treated with vehicle (ratio defined as 1 arbitrary unit, AU). For each set of culture conditions, the mean of triplicate ratios is indicated on the graph, together with the SD. [file 13045_2015_135_MOESM3_ESM.pdf]
