# Supplementary material for: Heat shock factor 1 is a potent therapeutic target for enhancing the efficacy of treatments for multiple myeloma with adverse prognosis
Source: J Hematol Oncol. 2015 Apr 23;8:40. doi: 10.1186/s13045-015-0135-3 (PMC4435646; doi:10.1186/s13045-015-0135-3)
Supplement: Additional file 4: — Interactions between the drugs analyzed by the combination index (CI) method. HMCLs were treated for 24 h with HSP90 or HSF1 inhibitors and with dexamethasone, bortezomib, or lenalidomide for additional 24 h at the indicated concentrations. Cell viability was then determined by MTT assay. CIs were calculated according to Chou [9]. CI < 1 indicates synergy; CI = 1, additive effect; CI > 1, antagonism (gray boxes). [file 13045_2015_135_MOESM4_ESM.docx]

**Additional File 4. Interactions between the drugs analyzed by the combination index (CI) method**

| Cell line | Drug (concentration) | KNK-437 (1 μM) | KNK-437 (10 μM) | 17-AAG (100 nM) |
| --- | --- | --- | --- | --- |
| L363 | Bortezomib (10 nM) | 0.71 | 0.32 | 0.69 |
|  | Dexamethasone (1 μM) | 0.62 | 0.76 | 1.2 |
|  | Lenalidomide (1 μM) | 1.24 | 1.49 | 1.61 |
| 8226 | Bortezomib (10 nM) | 0.59 | 0.72 | 0.79 |
|  | Dexamethasone (1 μM) | 0.87 | 0.67 | 0.87 |
|  | Lenalidomide (1 μM) | 1.38 | 4.19 | 3.40 |
| LP1 | Bortezomib (10 nM) | 0.39 | 0.28 | 1.57 |
|  | Dexamethasone (1 μM) | 0.69 | 0.81 | 1.33 |
|  | Lenalidomide (1 μM) | 2.19 | Not calculable | Not calculable |

HMCLs were treated for 24 h with HSP90 or HSF1 inhibitors and with dexamethasone, bortezomib or lenalidomide for additional 24 h at the indicated concentrations. Cell viability was then determined by MTT assay. CIs were calculated according to Chou [10]. CI < 1 indicates synergy; CI = 1, additive effect; CI > 1, antagonism (gray boxes).
