# Supplementary material for: Heat shock factor 1 is a potent therapeutic target for enhancing the efficacy of treatments for multiple myeloma with adverse prognosis
Source: J Hematol Oncol. 2015 Apr 23;8:40. doi: 10.1186/s13045-015-0135-3 (PMC4435646; doi:10.1186/s13045-015-0135-3)
Supplement: Additional file 5: — Clinical characteristics of MM/PCL patients. Samples were obtained from patients at diagnosis (D) or relapse (R); patients had multiple myeloma (MM), primary plasma cell leukemia (pPCL), or secondary plasma cell leukemia (sPCL). [file 13045_2015_135_MOESM5_ESM.docx]

**Additional File 5**

**Clinical characteristics of MM/PCL patients**

| Patient # |  |  | del(17)p | t(4;14) |
| --- | --- | --- | --- | --- |
| 1 | R | sPCL | + (97%) | - |
| 2 | D | MM | - | - |
| 3 | D | MM | + (90%) | - |
| 4 | D | MM | - | - |
| 5 | D | pPCL | + (97%) | - |

Samples were obtained form patients at diagnosis (D) or relapse (R); patients had multiple myeloma (MM), primary plasma cell leukemia (pPCL) or secondary plasma cell leukemia (sPCL).
