# Supplementary material for: Heat shock factor 1 is a potent therapeutic target for enhancing the efficacy of treatments for multiple myeloma with adverse prognosis
Source: J Hematol Oncol. 2015 Apr 23;8:40. doi: 10.1186/s13045-015-0135-3 (PMC4435646; doi:10.1186/s13045-015-0135-3)
Supplement: Additional file 6: — Additivity of bortezomib and KNK-347 co-treatment on MM primary samples. Primary cells were obtained from patients with MM or PCL. Purified CD13+ cells were cultured for 24 h and then treated with 5 nM bortezomib alone or in combination with 10 μM KNK-437. Cell death was determined as the percentage of CD138+ cells that have lost CD138. The percentage of dead cells directly measured (observed) and the percentage of dead cells calculated for an additive effect (expected) were not significantly different (p = 0.37, Wilcoxon matched-pairs signed-rank test). This signifies that the effect of the combination of both drugs was indeed additive. [file 13045_2015_135_MOESM6_ESM.docx]

**Additional File 6**

**Additivity of bortezomib and KNK-347 co-treatment on MM primary samples**

| Sample # | Bort 5 | KNK 10 | Bort+KNK | Expected value |
| --- | --- | --- | --- | --- |
| 1 | 16% | 5% | 15% | 21% |
| 2 | 19% | 8% | 63% | 27% |
| 3 | 39% | 2% | 51% | 41% |
| 4 | 23% | 2% | 51% | 41% |

Primary cells were obtained from patients with MM or PCL. Purified CD13+ cells were cultured for 24 h then treated with 5 nM bortezomib alone or in combination with 10 μM KNK-437. Cell death was determined as the percentage of CD138+ cells that have lost CD138. The percentage of dead cells directly measured (observed) and the percentage of dead cells calculated for an additive effect (expected) were not significantly different (p = 0.37, Wilcoxon matched-pairs signed-rank test). This signifies hat the effect of the combination of both drugs was indeed additive.
